# Anti-Group B Streptococcus antibody in infants born to mothers with human immunodeficiency virus (HIV) infection^☆^

**DOI:** 10.1016/j.vaccine.2014.12.025

**Published:** 2015-01-29

**Authors:** Kirsty Le Doare, Lauren Allen, Beate Kampmann, Paul Trafford Heath, Stephen Taylor, Anneke C. Hesseling, Andrew Gorringe, Christine Elizabeth Jones

**Affiliations:** a Wellcome Trust/Imperial Centre for Global Health Research/Department of Academic Paediatrics, Imperial College London, Norfolk Place, London W2 1NY, UK; b Paediatric Infectious Diseases Research Group, St George's, University of London, Cranmer Terrace, London SW17 0RE, UK; c Public Health England, Porton Down, Salisbury SP4 0JG, UK; d Medical Research Council, Atlantic Road, Fajara, Gambia; e Desmond Tutu TB Center, Department of Pediatrics and Child Health, Stellenbosch University, Cape Town, South Africa

**Keywords:** Antibody, Group B Streptococcus, HIV-exposed-uninfected infants, HIV, Immunity

## Abstract

**Background:**

HIV-exposed uninfected infants have increased infection risk and mortality compared to HIV-unexposed infants. HIV-exposed infants may be at increased risk of invasive GBS disease due to reduced maternal antibody against GBS.

**Methods:**

We quantified antibodies that bind to the surface of whole Group B Streptococcus (GBS) of serotypes Ia, Ib, II, III and V using novel flow cytometry assays in South African HIV-infected and non-infected mothers and their uninfected infants. Antibody-mediated complement C3b/iC3b deposition onto GBS of these serotypes was also quantified by a novel flow cytometry assay.

**Results:**

Geometric mean concentration (GMC) of both surface-binding anti-GBS antibody and antibody-mediated complement deposition onto GBS were reduced in HIV-infected women (*n* = 46) compared to HIV-uninfected women (*n* = 58) for ST1a (surface-binding: 19.3 vs 29.3; *p* = 0.003; complement deposition: 2.9 vs 5.3 SU/mL; *p* = 0.003), STIb (24.9 vs 47.6; *p* = 0.003; 2.6 vs 4.9 SU/mL; *p* = 0.003), STII (19.8 vs 50.0; *p* = 0.001; 3.1 vs 6.2 SU/mL; *p* = 0.001), STIII (27.8 vs 60.1; *p* = 0.001; 2.8 vs 5.3 SU/mL; *p* = 0.001) and STV (121.9 vs 185.6 SU/mL; *p* < 0.001) and in their infants for STIa (complement deposition 9.4 vs 27.0 SU/mL; *p* = 0.02), STIb (13.4 vs 24.5 SU/mL; *p* = 0.02), STII (14.6 vs 42.7 SU/mL; *p* = 0.03), STIII (26.6 vs 62.7 SU/mL; *p* = 0.03) and STV (90.4 vs 165.8 SU/mL; *p* = 0.04). Median transplacental transfer of antibody from HIV-infected women to their infants was reduced compared to HIV-uninfected women for GBS serotypes II (0.42 [IQR 0.22–0.59] vs 1.0 SU/mL [0.42–1.66]; *p* < 0.001), III (0.54 [0.31–1.03] vs 0.95 SU/mL [0.42–3.05], *p* = 0.05) and V (0.51 [0.28–0.79] vs 0.75 SU/mL [0.26–2.9], *p* = 0.04). The differences between infants remained significant at 16 weeks of age.

**Conclusions:**

Maternal HIV infection was associated with lower anti-GBS surface binding antibody concentration and antibody-mediated C3b/iC3b deposition onto GBS bacteria of serotypes Ia, Ib, II, III and V. This may render these infants more susceptible to early and late onset GBS disease.

## Highlights

• HIV-infected women have lower anti-GBS surface binding antibody concentrations than uninfected controls with reduced antibody-mediated deposition of complement C3b/iC3b onto GBS bacteria.
• HIV-exposed, uninfected infants have a lower concentration of antibody binding to the surface of GBS bacteria as well as a reduction in antibody-mediated deposition of complement C3b/iC3b onto the surface of GBS strains at birth compared to HIV-unexposed infants.
• As a result, HIV-exposed uninfected infants may be at increased risk of early and late onset GBS disease compared to unexposed infants.

## 1 Introduction

The increasing numbers of HIV-exposed infants who remain uninfected is testament to the success of prevention of mother-to-child transmission programs in resource-poor settings in the face of a high disease burden [1]. Nevertheless, this group of infants appears to suffer from increased rates of lower respiratory tract infection and meningitis compared to HIV-unexposed infants and up to 4-fold higher mortality in the first year of life [2–4]. *Streptococcus agalactiae* (group B streptococcus, GBS) is a leading cause of neonatal pneumonia, sepsis and meningitis in many countries and five serotypes (Ia, Ib, II, III and V) account for the majority of disease [5]. Published data show that not only is GBS carriage increased in HIV-infected pregnant women compared with HIV-uninfected mothers [6], but that HIV-exposed, uninfected infants also appear to have an increased risk of late-onset GBS disease compared to HIV-unexposed infants [7].

Prevention of GBS infection in infants and adults through immunization is a theoretically attainable goal. Maternal transfer of antibodies is thought to prevent newborn GBS disease [8] and GBS vaccines offer the potential to prevent disease in low-income settings where prenatal screening and intrapartum antibiotics are generally not feasible. A number of studies have demonstrated an association between the risk of developing invasive GBS disease in the newborn and maternal anti-capsular antibody levels [9–12] with opsonophagocytosis the likely effector mechanism [13–15]. However, maternal HIV status can influence the efficiency of transplacental antibody transfer, resulting in reduced maternally-derived specific antibody concentration in the infant, even where the infant is subsequently HIV uninfected [16–19]. Data regarding the persistence of anti-GBS antibodies in populations with high HIV-prevalence and their function in vitro would provide insight into their protective potential in different populations. Thus our objective was to determine the association between maternal HIV infection status and total IgG binding to the surface of GBS bacteria of serotypes Ia, Ib, II, II and V and antibody-mediated deposition of C3b/iC3b onto the surface of these GBS strains as a potential surrogate for opsonophagocytosis in sera obtained from mothers and infants.

## 2 Methods

### 2.1 Study setting, eligibility and study measures

Samples were collected from mothers and infants enrolled in a cohort study investigating the influence of maternal HIV and mycobacterial sensitization on infant immune responses to BCG vaccination carried out between 2009 and 2011 [20]. In brief, 109 pregnant women presenting to a Community Health Center in Khayelitsha, Western Cape Province, South Africa who delivered a healthy infant over 37 weeks gestation, weighing over 2.5 kg, knew the result of their HIV test and were willing to provide written informed consent for themselves and their infants were recruited to the original study. The subset represented all mothers/infants with available samples. A venous blood sample was collected from the mother and infant within 24 h of delivery. All infants had a further venous blood sample collected at 16 weeks. HIV-exposed infants had an HIV polymerase chain reaction (Amplicor HIV-a DNA kit, version 1.5; Roche Molecular Systems Inc., Branchburg, NJ) performed at ages 4 and 16 weeks. The study was approved by the Universities of Cape Town (382/2008) and Stellenbosch (N08/10/278), South Africa, and the National Health Service Research Ethics Committee, England (07/H0720/178).

In 2009, the HIV prevalence among women attending antenatal clinics in the area was 31% [21]. During the study period, the Prevention of Mother to Child Transmission program consisted of dual therapy for mothers and infants, starting with the administration of zidovudine at 28 or more weeks’ gestation, then zidovudine for 1 month to the infant and a single dose of nevirapine to both mother and infant. Mothers were eligible for highly active antiretroviral treatment if their CD4 count was less than 200 cells/μL. Exclusive infant feeding options were encouraged and mothers were provided with free formula for 6 months if they chose exclusive formula feeding.

### 2.2 Laboratory assays

The reproducibility of the assays was assessed between three different days and between three different operators with a panel of 18 sera. The assays had a coefficient of variation of less than 30%. Controls used were rabbit polyclonal sera (SSI) raised against serotypes Ia, Ib, II, III & V, and normal human donor sera.

#### 2.2.1 GBS isolates and growth conditions

Group B Streptococcus isolates used in this study were H092040676 (Serotype Ia), H090820125 (Serotype Ib), H090320548 (Serotype II), H092120162 (Serotype III) and H091780506 (Serotype V), which were kindly provided by Professor Androulla Efstratiou, Public Health England, Colindale. Strains are subsequently referred to by their serotype.

GBS isolates used in the antibody surface binding and complement deposition assays were grown in Todd Hewitt broth at 37 °C with shaking (200 rpm). Once OD_600nm_ 1.0 was reached, cultures were centrifuged at 3060 × *g* for 5 min to pellet the bacteria. The pellet was re-suspended in the same volume of phosphate buffered saline (PBS, pH 7.4, Severn Biotech, UK) containing 2% formaldehyde and incubated at room temperature for 1 h. Bacteria were then washed in PBS by three rounds of centrifugation at 3060 × *g* for 5 min and resuspended. The final cell pellet was resuspended in 1 mL PBS, which was stored at 4 °C before use.

#### 2.2.2 Sample preparation for antibody surface binding assay

Deposition of anti-GBS antibody onto the surface of whole GBS bacteria was measured on formaldehyde-fixed GBS using a flow cytometric assay performed in 96-well microtitration plates. Briefly, 2 μL of each test serum was added to 198 μL of serotype Ia, Ib, II, III or V GBS bacteria at 5.14 × 10^7^ CFU/mL in blocking buffer (1% BSA in PBS). This was incubated at 25 °C for 30 min with shaking (900 rpm), then pelleted. Supernatant was removed and the pellet washed twice with 200 μL of blocking buffer. Alexa Fluor^®^ 488 Goat Anti-Human IgG (H&L) (Life Technologies) (1:500) in blocking buffer was added and samples incubated for 20 min at 4 °C, before being washed twice more with blocking buffer.

#### 2.2.3 Sample preparation for antibody-mediated complement C3b/iC3b deposition assay

Antibody-mediated C3b/iC3b deposition on the surface of whole GBS bacteria was measured on the surface of formaldehyde-fixed GBS using a flow cytometric assay performed in 96-well microtitration plates. Briefly, 35 μL serotype Ia, Ib, II, III and V GBS bacteria at 5.14 × 10^7^ CFU/mL in blocking buffer (1% BSA in PBS) were added to 10 μL IgG-depleted human complement [22] and 5 μL of each test serum. Plates were incubated for 7.5 min at 37 °C with shaking (900 rpm) then pelleted. Supernatant was removed and the pellet washed once with 200 μL blocking buffer. Samples were resuspended in 200 μL blocking buffer containing 1:500 sheep anti-human C3c FITC (Abcam) and incubated as for the surface binding assay.

#### 2.2.4 Sample acquisition

Assays were analyzed using a Beckman Coulter Cyan flow cytometer equipped with a Cytek 96-well microtitre plate reader. Protocols were initially set-up to analyze profiles of bacterial events using bacteria-only control samples and the bacteria identified on the cytometer by the forward scatter (FS), measuring the size of the cell, and side scatter (SS), the granularity and internal structural complexity. An analysis ‘gate’ was drawn around the population of interest (single and diplococci) and a relevant histogram plot created to analyze the fluorescence of the bacterial population. For each sample, 10,000 individual events were analyzed for fluorescence and a horizontal gate was drawn to include 10% of the ‘no antibody’ control population. A fluorescence index (FI) was calculated for each sample, which involved the multiplication of the percentage of bacteria in the horizontal gate (%-gated), by the mean fluorescence of that population (X-mean). The final result for each test was expressed as the average FI of duplicate test samples minus the average FI of the bacteria and conjugate-only control. A standard unit (SU) measurement for each serum sample was then calculated by comparing to the serum FI response obtained with the positive control serum for each serotype which was given an arbitrary value of 1000 (kind gift from Professor Carol Baker, Baylor College of Medicine, Texas) to give a result in SU/mL.

### 2.3 Data management and statistical analysis

Sample size was determined for the cohort study; this sub-study had 80% power (*p* = 0.05) to investigate differences between antibody concentrations in HIV-exposed and HIV-unexposed infants of at least 30% with the pre-specified hypothesis that the concentrations would be lower in HIV-exposed infants. This assay is currently being formally compared with ELISA and opsonophagocytosis uptake assays in another study.

Statistical analyzes were completed using STATA version 12 (StataCorp 2013, La Jolla, CA) and GraphPad Prism version 6.0 (GraphPad Software Inc., La Jolla, CA). Due to the skewed distribution of the anti-GBS antibody concentrations, log transformations were required to conform to regression assumptions. A paired *t*-test was used to determine any difference in anti-GBS-antibody concentrations to each GBS serotype between birth and at 16 weeks. Comparison of infant anti-GBS antibody concentrations by maternal HIV status was calculated using the Wilcoxon rank sum test. Correlation of infant anti-GBS antibody concentration to each GBS serotype at birth and at 16 weeks was assessed using Spearman correlation. Multiple linear regression was used to ascertain the relationship between infant anti-GBS antibody concentration to each GBS serotype and the covariates of maternal age, infant sex and maternal HIV. In addition, multiple linear regression was used to explore the relationship between maternal anti-GBS antibody concentration to each serotype and the covariates maternal age, maternal HIV status. Placental transfer was defined as the ratio of infant-to-mother anti-GBS IgG concentration at birth. Missing data were excluded from analysis. Decline in antibody concentration between birth and 16 weeks was calculated using ratio of means.

## 3 Results

### 3.1 Participant characteristics

Samples were analyzed from 104 women (46 (44%) HIV-infected and 58 (56%) HIV-uninfected women) and from 100 infants born to this group of women (46 (46%) HIV-exposed and 54 (54%) HIV-unexposed infants). One mother–infant pair was excluded from the final analysis because HIV-infection in the infant was detected at 4 weeks of age and the infant was referred for rapid initiation of antiretroviral treatment. All other infants born to HIV-infected mothers were HIV-uninfected. All infants were born after 37 weeks gestation. Follow-up samples at 16 weeks were available for 93 infants (93%; 38 HIV-exposed and 55 HIV-unexposed) at a mean postnatal age of 16.4 weeks (SD = 1.7).

All HIV-infected women chose exclusive formula replacement feeding. The median (IQR) CD4 count among the HIV-infected women was 411.5 (293.9–604.0) cells/μL and the median (IQR) viral load was 800 (357–6000) copies/mL Seven women had CD4 counts of less than 200 cells/μL; three of these were taking highly active antiretroviral treatment at enrollment and four were referred to commence highly active antiretroviral treatment following delivery. Further characteristics of the study cohort have been described previously [20]. At the time of the study, local GBS disease incidence in Cape Town was reported at 0.67/1000 live births in a population with 24% of these infants being HIV-uninfected, born to HIV-infected mothers [23].

### 3.2 Maternal anti-GBS-antibody to GBS serotypes Ia, Ib, II, III and V

**Fig. 1:**
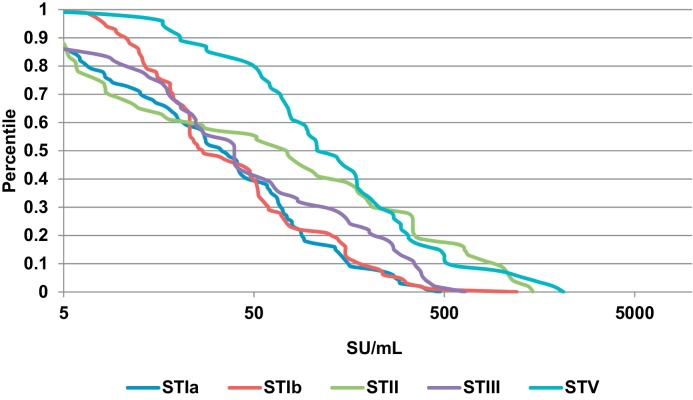
Reverse cumulative distribution curve of maternal anti-GBS IgG surface binding by serotype concentrations (SU/mL). Curve demonstrates the percentage of the total population with each antibody concentration for each of the five GBS serotypes. ST, serotype; SU/mL, standard units/mL

All maternal sera had measurable geometric mean concentrations (GMC) of antibody to at least one serotype. The distribution of mothers’ antibodies that bound to the surface of GBS serotypes Ia, Ib, II, III and V is shown in Fig. 1. The predominant surface binding anti-GBS-antibody was against serotype V, followed by II, III, Ib and Ia (Fig. 1).

**Fig. 2:**
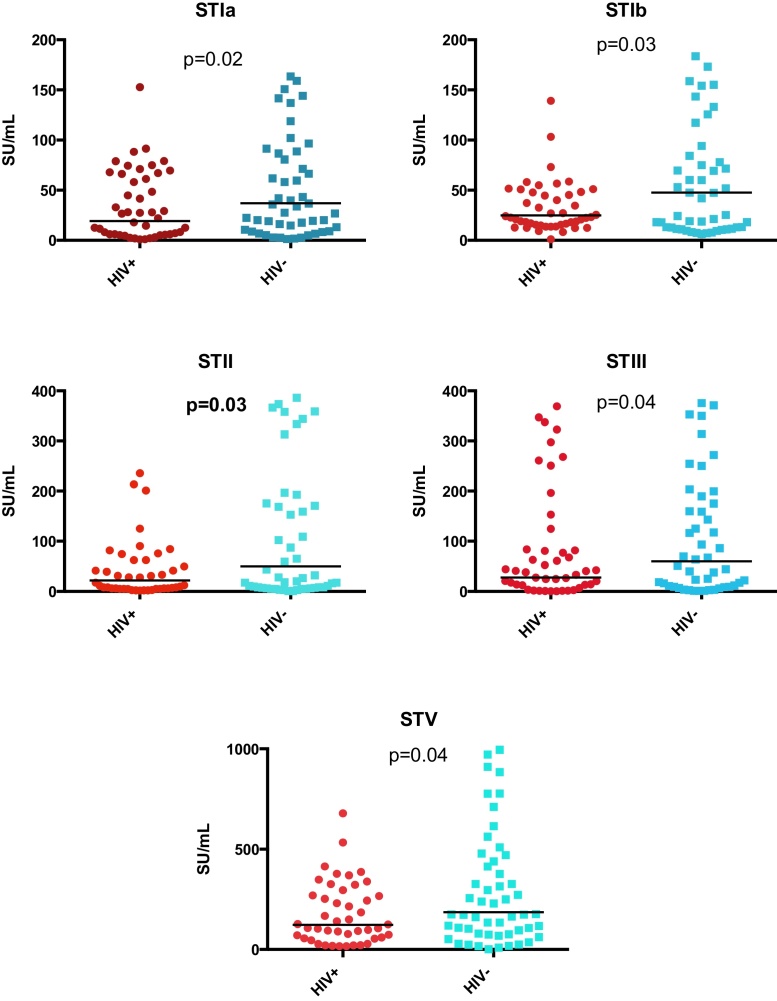
Maternal anti-GBS surface binding IgG to GBS serotypes Ia, Ib, II, III and V concentrations at delivery (SU/mL). ST indicates GBS serotype. HIV indicates human immunodeficiency virus. Horizontal lines indicate GMC response.

Women with HIV infection had significantly lower GMC antibody concentrations to all five serotypes at delivery compared to HIV-uninfected women (Fig. 2 and Table 1). There were no associations between CD4 count and viral load on anti-GBS antibody concentration by serotype in HIV-infected women.

The concentrations of antibody-mediated complement C3b/iC3b deposition by GBS serotype are shown in Table 2. The GMC of antibody-mediated C3b/iC3b deposition was significantly greater in HIV-uninfected than HIV-infected women for all serotypes. The GMC of antibody-mediated C3b/iC3b deposition ranged from 4.9 SU/mL (serotype III) to 6.17 SU/mL (serotype V) in HIV-uninfected women and from 2.6 SU/mL (serotype Ib) to 3.4 SU/mL (serotype V) in HIV-infected women.

### 3.3 Association of maternal HIV infection with placental transfer of anti-GBS antibody

There was a positive correlation between paired maternal and infant surface binding antibody concentrations for all serotypes (Ia, *r*^2^ = 0.63, *p* < 0.001; Ib, *r*^2^ = 0.71, *p* < 0.001; II, *r*^2^ = 0.17, *p* < 0.001; III, *r*^2^ = 0.35, *p* < 0.001; V, *r*^2^ = 0.16, *p* < 0.001). HIV-infected women had significant reductions in placental transfer of anti-GBS serotypes II and V compared with HIV-uninfected women (Table 3). There was no association between CD4 count or viral load on transplacental transfer ratios.

### 3.4 Infant anti-GBS-antibody by serotype at birth to GBS serotypes Ia, Ib, II, III and V

**Fig. 3:**
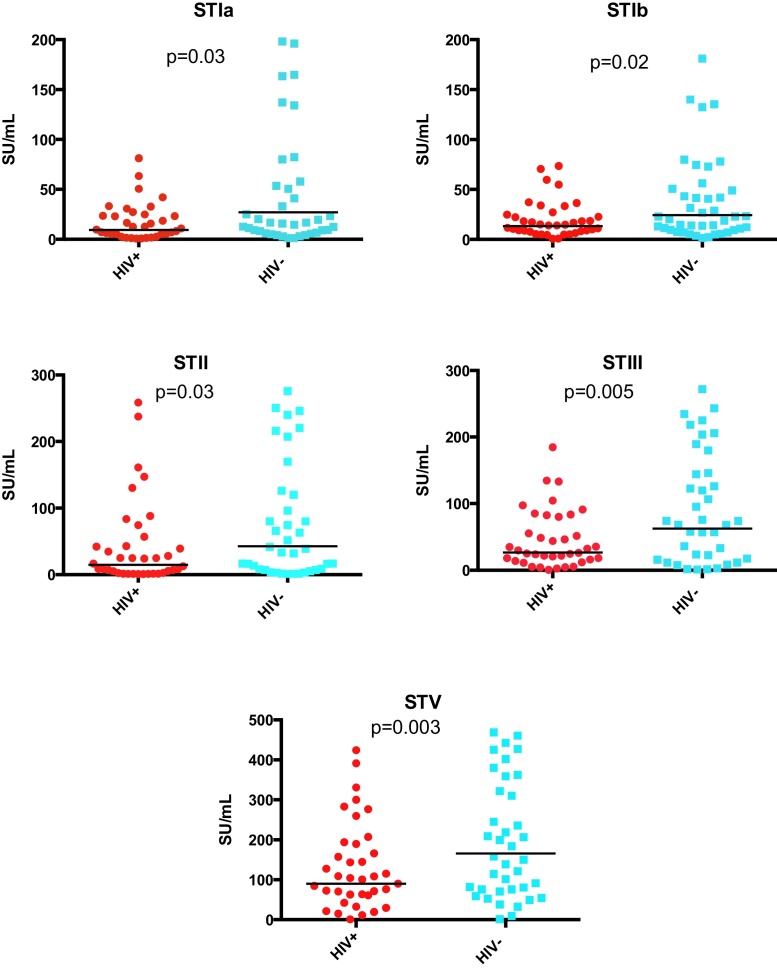
Infant anti-GBS IgG serotype specific antibody concentrations (SU/mL). ST indicates GBS serotype. HIV indicates human immunodeficiency virus. Horizontal lines indicate GMC.

HIV-exposed uninfected infants had significantly lower GMCs of anti-GBS surface binding antibodies to all serotypes than unexposed infants (Fig. 3 and Table 1). In a multiple regression model maternal HIV-status remained significant (*p* < 0.05).

However, there were no significant associations found between infant anti-GBS-antibody concentrations to each serotype and maternal age or infant sex.

Significantly lower GMC of antibody-mediated C3b/iC3b deposition was noted amongst HIV-exposed infants compared to HIV-unexposed infants for all serotypes (Table 2). Notably, the GMC of antibody-mediated C3b/iC3b deposition was higher in infants than in their mothers for all serotypes.

### 3.5 Infant anti-GBS antibody at 16 weeks of age

Anti-GBS surface binding antibody GMCs remained significantly lower at 16 weeks in HIV-exposed uninfected infants compared to unexposed infants for all serotypes (Table 1). HIV-exposed infants had significantly slower median rate of antibody decline compared to HIV-unexposed infants at 16 weeks compared to birth to STII (ratio of decline 1.03 [IQR −0.35 to 4.6] vs 0.21 SU/mL [−0.62 to 0.54], *p* = 0.04) and STIII (1.53 [−0.21 to 5.36] vs −0.09 SU/mL (−0.60 to 0.93), *p* = 0.03) but not to ST1a (−0.12 [IQR −0.64 to 0.13] vs −0.04 [−0.39 to 0.45]), ST1b (−0.52 [−0.65 to 0.46] vs −0.36 [−0.18 to 0.74]) or STV (−0.40 [−0.61 to 2.9] vs 0.14 [−0.52 to 2.7]).

## 4 Discussion

Our findings demonstrate that total surface binding and functional maternal antibody concentration to each of the GBS serotypes (Ia, Ib, II, III and V) is lower at delivery in HIV-infected women compared to their HIV-uninfected peers. Further, for three of the five serotypes (II, III, V) the transplacental transfer ratio was significantly lower from HIV-infected women to their uninfected infants. It follows therefore that infants born to HIV-infected women had lower anti-GBS surface binding antibody concentrations at birth for all serotypes; this difference was still evident at 16 weeks of age. The implication of our findings is that HIV-exposed infants may be more vulnerable to early and late onset GBS disease than infants born to HIV-uninfected mothers.

Rectovaginal colonization with GBS is more frequent in HIV-infected women compared to HIV-uninfected women [6,24]. Maternal colonization is the major risk factor for infant acquisition at birth and for early onset GBS disease. Thus infants born to HIV infected mothers may have both increased susceptibility and increased exposure. It is generally understood that maternal colonization stimulates maternal immunity; thus the finding of lower maternal antibody concentrations despite higher colonization rates suggests impairment of anti-GBS-specific antibody production. This is also seen with polysaccharide-specific antibody production in HIV-infected adults, such as in pneumococcal disease [25]. The fact that HIV-infected women have higher rates of GBS colonization than HIV-uninfected women may mean that antibody production secondary to colonization with GBS is deficient in the context of maternal HIV-infection increasing the risk of invasive GBS disease in HIV-exposed infants.

It is difficult to correlate our anti-GBS antibody results with what is known regarding maternal colonization and infant GBS disease in this South African population. However, the highest concentrations of antibodies in women at delivery were to STV and III bacteria. The latter is consistent with the known predominance of STIII among carried strains in South African women (37%) but STV is carried by a minority of women (10%) [24,26]. In our cohort the lowest levels of antibody were to STIa bacteria, which is consistent with an increased incidence of disease due to this serotype [26]. Our findings may suggest differences in the immune response to different colonizing serotypes.

It is likely that lower maternal antibody concentrations and altered transplacental transfer of antibody in HIV infection play a role in the increased susceptibility to infectious diseases in infancy [19,27], but there is a lack of data on the impact of maternal HIV infection on neonatal infections in general, including GBS infections [28]. Infant anti-GBS surface binding antibody by serotype remains lower at 16 weeks in HIV-exposed, uninfected infants, who might therefore be more vulnerable to late onset GBS disease. This corresponds to recent data from Cape Town which suggest that 56% of GBS disease occurred after 7 days of life in a population where one in four infants were HIV-exposed [23]. Apart from the reduced passive immunity provided by maternal antibody in the context of maternal HIV infection it might be that there are differences in the immune response to infection of HIV-exposed, uninfected infants secondary to *in utero* HIV exposure, as some data suggest that these infants have altered lymphocyte differentiation and function despite remaining HIV-uninfected [29,30]. However, how this corresponds to clinical infection in the neonatal period is not known.

Our findings are consistent with studies that demonstrate the higher risk of infectious morbidity and mortality in HIV-exposed infants compared to HIV-unexposed infants [31–33]. Recent South African studies demonstrated a high prevalence of neonatal GBS disease in a population with a high HIV burden [23,24,34]. A Study from Malawi also demonstrated high rates of early and late onset GBS infection in a population with high HIV prevalence [35]. In contrast, one study from the USA did not find evidence of increased susceptibility to GBS infection in HIV-exposed, uninfected infants suggesting either regional screening and treatment differences for both HIV infection and GBS carriage or population-based differences in late onset GBS disease that might be unrelated to HIV-status [36].

Our study has some limitations. Firstly, we did not assess colonization in the mothers participating in the study and thus were unable to correlate antibody responses to maternal colonization. Whilst no infant developed early or late onset sepsis or meningitis during the study, our sample size was also too small to detect this as a valid endpoint and this was not the aim of the study.

The use of two novel flow cytometric assays that quantify antibody binding to the surface of GBS bacteria and antibody-mediated C3b/iC3b deposition on the bacterial surface may provide a more complete assessment of antibody function than the measurement of anti-polysaccharide IgG alone. However, the response measured is not wholly serotype-specific as part of the antibody binding is to cell wall proteins as well as the polysaccharide capsule. Despite this, we see a serotype-specific response with a clear differentiation between the distribution of antibody binding to the different serotypes.

The potential of a trivalent maternal GBS vaccine encompassing the major disease-causing serotypes (Ia, Ib, III) [8] to protect against neonatal disease remains promising. However, it is vital that ongoing evidence to support its use in vulnerable populations with high HIV-prevalence is collected in order to support immunization strategies in Sub-Saharan Africa.

## Figures and Tables

**Table 1:** Geometric mean surface binding antibody concentrations (95% confidence intervals) in HIV-infected and HIV-uninfected mothers and their infants at birth and at 16 weeks of age.

	STIa	STIb	STII	STIII	STV
HIV+ mother	19.3 (12.7–29.3)	24.9 (19.6–31.6)	21.8 (13.4–35.7)	27.8 (15.9–48.6)	121.9 (88.0–169.0)
HIV− mother	37.0 (24.8–55.2)	47.6 (33.3–68.1)	50.0 (28.7–87.0)	60.1 (36.0–100.5)	185.6 (126.4–272.4)
HIV-exposed infants (birth)	9.4 (6.1–14.5)	13.4 (9.6–18.7)	14.6 (8.1–26.3)	26.6 (17.8–39.8)	90.4 (61.16–133.6)
HIV-unexposed infants (birth)	27.0 (16.5–44.3)	24.5 (16.4–36.5)	42.7 (23.4–77.8)	62.7 (38.1–103.1)	165.8 (112.0–245.4)
HIV-exposed infants (16 w)	7.0 (3.8–13.0)	8.7 (6.3–12.1)	17.6 (8.5–36.3)	48.0 (28.3–81.3)	68.54 (38.0–123.6)
HIV-unexposed infants (16 w)	21.6 (13.6–34.2)	14.9 (10.6–21.1)	68.3 (38.8–120.3)	97.4 (63.0–150.7)	180.2 (106.3–305.4)

STIa, GBS serotype Ia; STIb, GBS serotype Ib; STII, GBS serotype STII; STIII, GBS serotype III; STV, GBS serotype V; HIV+, HIV infected; HIV−, HIV uninfected; 16 w, 16 weeks of age.

**Table 2:** Geometric mean concentration of antibody-mediated C3b/iC3b deposition (95% confidence intervals) on GBS serotypes Ia, Ib, II, III and V in HIV-infected and HIV-uninfected mothers and their infants at birth.

	STIa	*p* value	STIb	*p* value	STII	*p* value	STIII	*p* value	STV	*p* value
HIV+ mother	2.9 (2.3–3.6)		2.6 (1.9–3.2)		3.1 (2.4–3.9)		2.8 (2.2–3.6)		3.4 (2.8–4.2)	
HIV− mother	5.3 (4.6–6.2)	0.003	4.9 (4.2–5.4)	0.003	6.2 (5.6–6.8)	0.001	5.3 (4.5–6.2)	0.001	6.2 (4.9–6.9)	<0.0001
HIV-unexposed infants	6.1 (5.9–6.3)		5.9 (4.6–6.2)		6.9 (5.6–7.2)		6.6 (6.4–6.7)		6.3 (5.8–6.9)	
HIV-exposed infants	3.8 (3.0–4.8)	0.02	3.1 (3.0–4.1)	0.02	4.2 (3.9–4.9)	0.02	3.9 (3.1–5.0)	0.03	4.2 (3.3–5.4)	0.04

STIa, GBS serotype Ia; STIb, GBS serotype Ib; STII, GBS serotype STII; STIII, GBS serotype III; STV, GBS serotype V; HIV+, HIV infected; HIV−, HIV uninfected.

**Table 3:** Influence of maternal HIV-infection on transplacental transfer of anti-GBS surface binding antibody to GBS serotypes Ia, Ib, II, III and V.

GBS serotype	HIV-infected mothers: infants (IQR)	HIV-uninfected mothers: infants (IQR)	% Reduction in HIV-infected	*p* value
STIa	0.66 (0.34–0.99)	0.6 (0.39–0.96)	0%	0.86
STIb	0.48 (0.17–0.88)	0.52 (0.32–0.78)	8%	0.48
STII	0.42 (0.22–0.59)	1.0 (0.42–1.66)	58%	<0.001
STIII	0.54 (0.31–1.03)	0.95 (0.4–3.05)	43%	0.05
STV	0.51 (0.28–0.79)	0.75 (0.26–2.9)	32%	0.04

STIa, GBS serotype Ia; STIb, GBS serotype Ib; STII, GBS serotype STII; STIII, GBS serotype III; STV, GBS serotype V; IQR, interquartile range; HIV+, HIV infected, HIV−, HIV uninfected; 16 w, 16 weeks of age.

## Appendix A Supplementary data

Legend: Flowcytometry plot demonstrating bacteria only control, complement only control and positive test serum for serotype Ib. Top box - black and white, bottom box in colour
