# Supplementary material for: Anti-Group B Streptococcus antibody in infants born to mothers with human immunodeficiency virus (HIV) infection
Source: Vaccine. 2015 Jan 29;33(5):621–7. doi: 10.1016/j.vaccine.2014.12.025 (PMC4315133; doi:10.1016/j.vaccine.2014.12.025)
Supplement: Supplementary file 1 [file mmc1.pptx]

## Slide 1
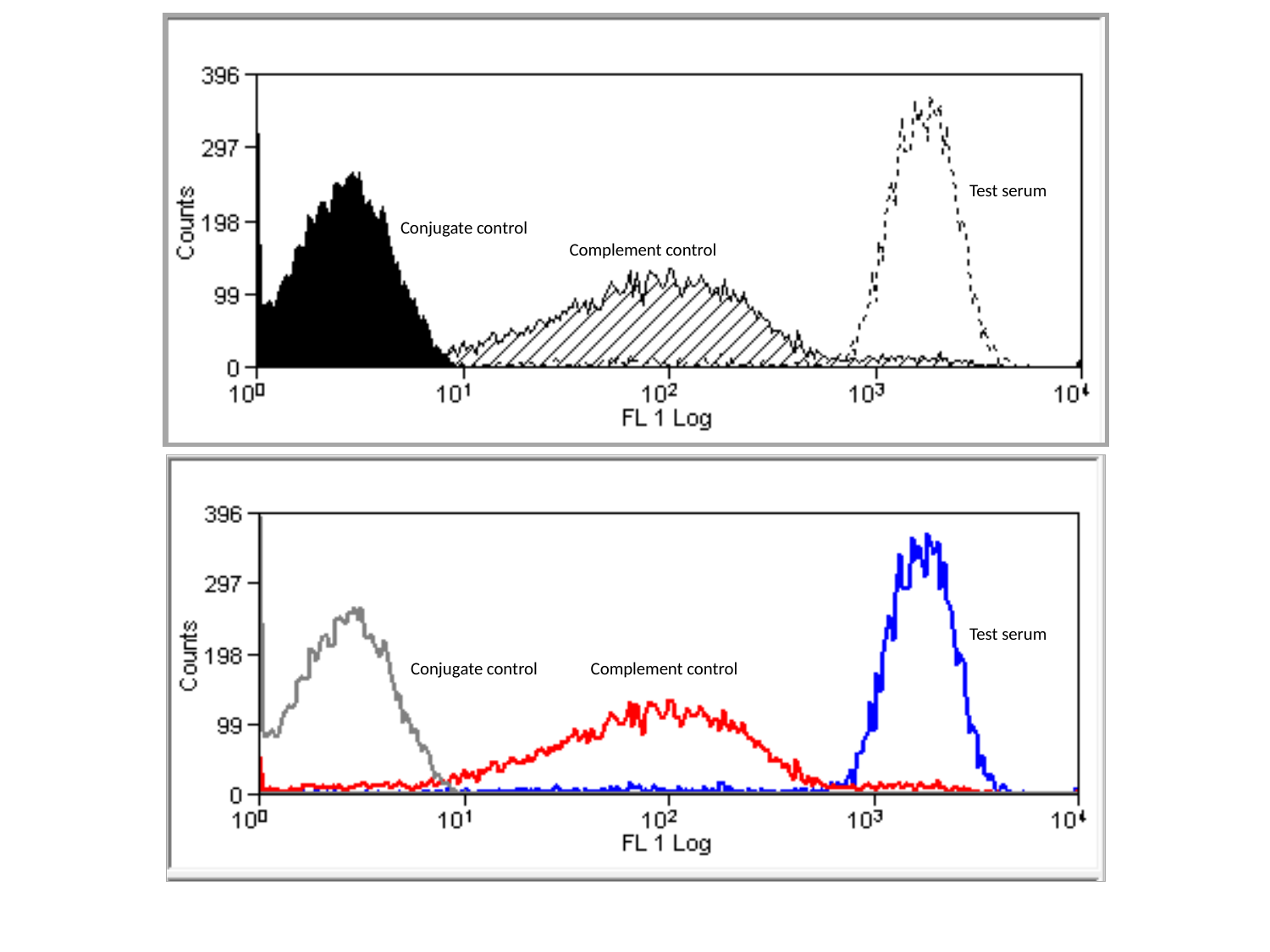

Test serum
Conjugate control
Complement control
Test serum
Conjugate control
Complement control
